# Supplementary material for: Influence of ABO Locus on PFA-100 Collagen-ADP Closure Time Is Not Totally Dependent on the Von Willebrand Factor. Results of a GWAS on GAIT-2 Project Phenotypes
Source: Int J Mol Sci. 2019 Jun 30;20(13):3221. doi: 10.3390/ijms20133221 (PMC6651679; doi:10.3390/ijms20133221)
Supplement: Supplementary file 1 [file ijms-20-03221-s001.pdf]

Supplementary Table S1. Description of the 70 significant SNPs associated with PFA-100 phenotypes: position in the *ABO* gene (chromosome 9), location, and significance levels. For collagen-ADP only, the same parameters after adjusting for von Willebrand factor and for coagulation factor VIII.

				Association with Col-Epi p-value	Association with Col-ADP p-value		
SNP name	Position (bp)	Location	MAF	Non-adjusted	Non-adjusted	Adjusted for VWF	Adjusted for FVIII
rs8176719	136132909	coding, 5-UTR, intron	0.459	1.88^{-9}	5.21^{-14}		
rs687621	136137066	intron	0.432	3.50^{-9}	1.50^{-15}	1.85^{-10}	6.58^{-11}
rs687289	136137107	intron	0.433	5.72^{-9}	1.93^{-15}	1.96^{-10}	7.12^{-11}
rs8176685	136138766	intron	0.310	5.80^{-9}	1.10^{-12}	1.72^{-8}	6.51^{-9}
rs657152	136139266	intron	0.460	1.52^{-9}	6.09^{-15}	4.10^{-10}	1.89^{-10}
rs149092047	136139908	intron	0.349		3.32^{-10}		
rs2519093	136141871	intron	0.312	6.01^{-9}	1.16^{-12}	1.66^{-8}	6.27^{-9}
rs514659	136142204	intron	0.433	1.04^{-8}	6.04^{-15}		2.02^{-10}
rs644234	136142218	intron	0.460	2.18^{-9}	1.43^{-14}	4.86^{-10}	3.47^{-10}
rs643434	136142356	intron	0.460	2.18^{-9}	1.43^{-14}	7.40^{-10}	3.47^{-10}
rs543040	136143001	intron	0.433	1.04^{-8}	6.10^{-15}	7.40^{-10}	2.01^{-10}
rs613534	136143121	intron	0.460	2.18^{-9}	1.43^{-14}	4.88^{-10}	3.47^{-10}
rs543968	136143122	intron	0.460	2.18^{-9}	1.43^{-14}	7.40^{-10}	3.47^{-10}
rs544873	136143213	intron	0.460	2.18^{-9}	1.43^{-14}	7.40^{-10}	3.48^{-10}
rs545971	136143373	intron	0.433	1.04^{-8}	6.03^{-15}	7.40^{-10}	2.02^{-10}
rs612169	136143443	intron	0.433	1.04^{-8}	6.05^{-15}	4.85^{-10}	2.02^{-10}
rs597988	136144285	intron	0.407	1.40^{-8}	1.67^{-15}	4.87^{-10}	5.00^{-11}
rs597974	136144298	intron	0.406	1.45^{-8}	4.08^{-15}	1.55^{-10}	1.02^{-10}
rs140114497	136144309	intron	0.394		8.12^{-14}	3.26^{-10}	1.37^{-9}
rs576125	136144310	intron	0.394		8.12^{-14}	4.87^{-9}	1.37^{-9}
rs8176663	136144428	intron	0.433	1.04^{-8}	6.05^{-15}	4.87^{-9}	2.02^{-10}
rs491626	136144874	intron	0.433	1.04^{-8}	6.05^{-15}	4.87^{-10}	2.02^{-10}

rs492488	136144961	intron	0.433	1.04^{-8}	6.05^{-15}	4.87^{-10}	2.02^{-10}
rs493246	136144995	intron	0.433	1.04^{-8}	6.06^{-15}	4.87^{-10}	2.02^{-10}
rs494242	136145119	intron	0.460	2.17^{-9}	1.43^{-14}	4.87^{-10}	3.48^{-10}
rs495203	136145241	intron	0.433	1.04^{-8}	6.06^{-15}	7.42^{-10}	2.02^{-10}
rs200700167	136145404	intron	0.368		1.74^{-9}	4.88^{-10}	
rs200533593	136145419	intron	0.341	3.66^{-8}	1.93^{-11}		
rs201298979	136145425	intron	0.305		5.28^{-9}		
rs9411378	136145426	intron	0.317		4.42^{-8}		
rs582118	136145472	intron	0.433	1.04^{-8}	6.06^{-15}	4.88^{-10}	2.02^{-10}
rs582094	136145485	intron	0.433	1.04^{-8}	6.06^{-15}	4.88^{-10}	2.02^{-10}
rs34357864	136145908	intron	0.429	1.19^{-8}	4.10^{-15}	4.17^{-10}	1.68^{-10}
rs2769071	136145975	intron	0.429	1.18^{-8}	4.28^{-15}	4.32^{-10}	1.74^{-10}
rs677355	136146047	intron	0.423		1.03^{-14}	6.49^{-10}	3.08^{-10}
rs200738971	136146062	intron	0.423		1.03^{-14}	6.49^{-10}	3.08^{-10}
rs59602812	136146069	intron	0.423		1.03^{-14}	6.49^{-10}	3.08^{-10}
rs676996	136146078	intron	0.423		1.03^{-14}	6.49^{-10}	3.08^{-10}
rs676457	136146228	intron	0.433	1.05^{-8}	6.12^{-15}	4.93^{-10}	2.04^{-10}
rs527210	136146432	intron	0.433	1.05^{-8}	6.12^{-15}	4.93^{-10}	2.04^{-10}
rs139840563	136146449	intron	0.412		6.17^{-14}	1.09^{-9}	9.55^{-10}
rs550057	136146598	intron	0.351		5.93^{-10}		
rs674302	136146665	intron	0.433	1.04^{-8}	6.06^{-15}	4.88^{-10}	2.02^{-10}
rs554833	136147161	intron	0.433	1.03^{-8}	6.14^{-15}	4.97^{-10}	2.04^{-10}
rs660340	136147554	intron	0.389		8.47^{-9}		
rs581107	136147703	intron	0.389		8.61^{-9}		
rs659104	136147824	intron	0.389		8.49^{-9}		
rs647800	136148001	intron	0.362		7.40^{-9}		
rs473533	136148036	intron	0.389		8.50^{-9}		
rs475419	136148232	intron	0.389		8.52^{-9}		

rs476410	136148369	intron	0.389		8.53^{-9}		
rs645982	136148410	intron	0.389		8.54^{-9}		
rs8176646	136149096	intron	0.460	2.35^{-9}	2.25^{-14}	1.07^{-9}	4.00^{-10}
rs116552240	136149099	intron	0.460	2.38^{-9}	2.22^{-14}	1.06^{-9}	3.95^{-10}
rs505922	136149230	intron	0.433	1.12^{-8}	9.68^{-15}	7.20^{-10}	2.39^{-10}
rs507666	136149400	intron	0.313	6.36^{-9}	2.29^{-12}	3.19^{-8}	1.04^{-8}
rs529565	136149501	intron	0.434	1.00^{-8}	1.86^{-14}	1.26^{-9}	3.91^{-10}
rs630510	136149582	intron	0.407		8.75^{-10}		
rs146518861	136149710	intron	0.310	9.30^{-9}	9.17^{-13}	1.57^{-8}	5.97^{-9}
rs630014	136149723	intron	0.407		8.74^{-10}		
rs532436	136149831	intron	0.312	5.80^{-9}	1.50^{-12}	2.18^{-8}	7.61^{-9}
rs616154	136150467	promoter, intron	0.413		4.71^{-10}		
rs559723	136150485	promoter, intron	0.413		4.71^{-10}		
rs600038	136151807	promoter, intergenic	0.340	1.70^{-9}	5.70^{-12}	4.36^{-8}	1.57^{-8}
rs651007	136153876	intergenic	0.340	2.32^{-9}	8.02^{-12}	4.84^{-8}	
rs579459	136154169	intergenic	0.338	1.35^{-9}	3.75^{-12}	2.58^{-8}	
rs649129	136154305	intergenic	0.340	2.19^{-9}	6.88^{-12}	4.22^{-8}	
rs495828	136154868	intergenic	0.340	2.28^{-9}	7.71^{-12}	4.69^{-8}	
rs635634	136155001	intergenic	0.312	4.74^{-9}	9.69^{-13}	1.33^{-8}	4.74^{-9}
rs633862	136155445	intergenic	0.391		2.41^{-9}		

The Genome Reference release used was GRCh37/hg19.p13

SNP = single nucleotide polymorphism; MAF = minor allele frequency; Col-Epi = collagen-epinephrine closure time; Col-ADP = collagen-ADP closure time; VWF = von Willebrand factor antigen; FVIII = coagulant factor VIII activity.

Col-Epi was adjusted for VWF, FVIII and *ABO* genotype; in all of these adjustments all the SNPs lost their genome-wide significance (p-value < 5×10^{-8}), so they do not appear in the table. Col-ADP was adjusted for VWF, FVIII and *ABO* genotype; in the VWF and FVIII adjustments, the number of significant SNPs was reduced as well as its significance, and, in the *ABO* genotype adjustment, all the SNPs lost their statistical significance, so they do not appear in the table.
